# The role of word form in gender processing during lexical access: A theoretical review and novel proposal in language comprehension

**DOI:** 10.3758/s13423-023-02426-8

**Published:** 2024-02-21

**Authors:** A. R. Sá-Leite, S. Lago

**Affiliations:** 1 Institut für Romanische Sprachen und Literaturen, Goethe University Frankfurt, Frankfurt, Germany; 2 Cognitive Processes and Behaviour Research Group, Department of Social Psychology, Basic Psychology and Methodology, University of Santiago de Compostela, 15782 Santiago de Compostela, Spain

**Keywords:** Grammatical gender, Gender transparency, AUSTRAL, Comprehension, Systematic review

## Abstract

In contrast to language production, there are few comprehension models of the representation and use of grammatical gender in long-term memory. To bridge this gap, we conducted a systematic review of empirical studies on the role of gender-form regularities in the recognition of nouns in isolation and within sentences. The results of a final sample of 40 studies suggest that there are two routes for the retrieval of gender during real-time comprehension: a form-based route and a lexical-based route. Our review indicates that the use of these routes depends on the degree of gender transparency of the language and the degree of overtness of the experimental paradigm. To accommodate these findings, we incorporate a dual-route mechanism within a general model of lexical access in comprehension, the AUSTRAL (Activation Using Structurally Tiered Representations and Lemmas) model, and identify directions for future research.

## Introduction

The study of grammatical gender reveals an interesting puzzle across languages. For instance, while a Spanish speaker considers the noun “screw” to be masculine (“tornillo”), a German speaker considers it to be feminine (“Schraube”). Interestingly, “tornillo” ends in “-o”, a productive nominal ending for the masculine value in Spanish. Meanwhile, “Schraube” ends in “-e”, which is a common pseudo-suffix for the feminine value in German (Wegener, 1985). Indeed, many languages display regularities between the distributional patterns of grammatical gender and the ortho-phonological form of nouns, so that some word-form patterns correlate with gender. This link between noun form and grammatical gender is known as “gender transparency” (Bates et al., 1995). This study examines whether gender transparency has a role in the cognitive recovery of the gender of a noun during real-time language comprehension.

## The concepts of grammatical gender and gender transparency

In languages with grammatical gender, such as Hebrew, Dutch, German, and Spanish, gender values are inherent features of nouns. When nouns are encountered, comprehenders encode not only their meaning and form, but also a series of grammatical characteristics such as their word class, number, and gender. These features allow the performance of syntactic operations during real-time comprehension. Therefore, the processing of gender occurs every time a noun is produced or comprehended, especially – but not only – when computing syntactic operations like noun-determiner or noun-adjective agreement (Paolieri et al., 2010, 2011; Sá-Leite et al., 2019, 2021). For instance, in the Spanish sentence "Ese _(masculine)_ es mi libro _(masculine)_" ("That one is my book"), the noun “book” determines the gender marking of the demonstrative “Ese” (masculine “that”; compare with the feminine “that”, i.e., “esa”). Some other nouns can be marked for gender, but these are said to have natural or semantic gender: Their gender is related to the biological or perceived sex of the referent, for example, “chico/chica” (boy/girl) in Spanish. Central to the present review is the relationship between non-semantic or grammatical gender (henceforth: gender) and noun form.

Depending on the predictability of gender from a noun’s form, we can classify nouns into three categories: regular, irregular, and ambiguous (Bates et al., 1995). *Regular nouns* display regularities associated with gender, such as the suffix “-o” for a masculine noun in Spanish (e.g., “tornillo”). *Irregular nouns* display a seemingly typical regular pattern but they are actually of the opposite gender, for example the noun “radio” in Spanish, which is feminine despite ending in “-o.” Finally, *ambiguous nouns* are those whose gender value cannot be predicted from their form, for example nouns ending in “-e” in Spanish.

Based on the predictability of gender from cues internal to nouns, Kupisch et al. (2018) established a continuum of gender transparency across languages. Languages like Dutch or Norwegian are classified as opaque because gender values can be hardly predicted from the noun’s form (Kupisch et al., 2022; Popov & Bastiaanse, 2018; Rodina & Westergaard, 2017a, b). Languages like Spanish, Italian, or Portuguese are considered highly transparent, because they display identifiable form regularities in most nouns, prominently “-o” for masculine nouns and “-a” for feminine nouns (Bates et al., 1995; Garrido-Pozú, 2022; Harris, 1991; Sá-Leite, 2021). Languages like Hebrew, Bulgarian, or Russian are considered moderately transparent (Gollan & Frost, 2001; Ivanova-Sullivan & Sekerina, 2019; Rodina & Westergaard, 2015). French and German are even more opaque. In these languages, multiple gender regularities exist. For instance, Köpcke & Zubin (1983) identified more than 44 gender-form regularities in monomorphemic nouns in German (e.g., “-en” or “-el” for masculine), and many others in more complex nouns (e.g., suffixes “-ung” or “-keit” for feminine nouns). Yet, there are many exceptions to these regularities, which make gender harder to predict compared to languages located on the more transparent side of the continuum.

## A double mechanism of gender processing

It is debated in the literature how gender values are recovered during lexical access to nouns (Sá-Leite et al., 2022; Wang & Schiller, 2019). There are few proposals about lexical access that are explicit with regard to how gender is stored and accessed in the mental lexicon. Under most models of word production, when a noun is accessed, the gender information associated with its whole lexical entry is recovered from memory (Caramazza, 1997; Levelt et al., 1999). However, many studies have shown that in non-opaque languages, comprehenders can access a noun’s gender from regularities in its form – without necessarily having to retrieve its entire lexical entry – for instance by assigning gender to pseudo-nouns that display such regularities (e.g., Arias-Trejo et al., 2013; Hohlfeld, 2006; Ogneva, 2023; Tucker et al., 1968). These findings have motivated proposals of a double route for gender retrieval during lexical access.

A double route for gender retrieval was first put forward by Taft & Meunier (1998), who found that French speakers were faster and more accurate when reporting the gender of regular versus ambiguous nouns. This effect occurred regardless of noun frequency: Participants had faster gender decisions for high- than for low-frequency nouns, but both types of nouns benefited similarly from the presence of form regularities. To account for these findings, the researchers initially proposed that gender was retrieved from lexical information stored within the noun (a lexical route), and that orthography was only used later to verify gender retrieval (a sub-lexical route). However, they proposed that there was little reason to consult form-based information if gender could be successfully determined based on lexical information alone – doing so might even constitute a distraction with irregular and ambiguous words. For this reason, they opted for an explanation based on neural network models (Seidenberg & McClelland, 1989), whereby form-based factors affect the process of activating the relevant lexical information. According to this account, gender retrieval is based on lexical information, but the ease of activating lexical information depends on the systematicity of the relationship between form and function.

Later on, Gollan & Frost (2001) proposed the Dual-Route model of gender retrieval, which gained popularity among researchers interested in gender transparency. ﻿They developed the model to account for a series of results in Hebrew, a moderately transparent language with two gender values, masculine and feminine. Gollan & Frost (2001) conducted gender categorization and grammaticality judgment tasks. In gender-categorization tasks, participants showed faster categorization responses with regular versus ambiguous nouns. In grammaticality judgment tasks, participants were faster and more accurate detecting agreement violations in sentences with regular versus irregular nouns. These results were interpreted as suggesting that gender was not simply recovered from memory – in association with the full-form representation of a word – but was also accessed through form-based gender cues. The authors hence proposed the Dual-Route model with a lexical and a form-based route of gender retrieval, paralleling Taft & Meunier’s (1998) proposal.

The recovery of gender through a lexical route is supported by evidence from tip-of-the-tongue states and language impairments (e.g., anomia and aphasia), which show that speakers can access the gender of nouns without accessing their form (Badecker et al., 1995; Caramazza & Miozzo, 1997; Henaff-Gonon et al., 1989; Miozzo & Caramazza, 1997a, b; Vigliocco et al., 1997). In unimpaired speakers, the open question is under what circumstances a form-based route is used. According to Gollan & Frost (2001), the lexical route should be used with all types of nouns, but the form-based route should mostly be used with regular nouns or under special conditions – for example, when a task explicitly requires participants to categorize nouns, or in agreement-violation paradigms. Thus, the authors considered the simultaneous involvement of the form-based and lexical routes, but were more inclined to consider the form-based route as a post-lexical checking mechanism.

While the proposals of Taft & Meunier (1998) and Gollan & Frost (2001) were empirically supported by experiments in French and Hebrew, their cross-linguistic generalizability is unclear, especially with more transparent languages. In these languages, effects related to gender transparency do not seem restricted to gender-categorization tasks or agreement violation designs, and they have been shown to appear early during lexical access in paradigms with more fine-grained temporal resolution (e.g., electroencephalography), which is at odds with the post-lexical status of a form-based route (Caffarra et al., 2014, 2015; De Martino et al., 2011). The current study reviews these cross-linguistic findings in order to revisit proposals about the use of a dual mechanism of gender processing in comprehension.

## The current study

The current study has two goals. The first is to carry out a systematic review of the literature on gender regularities in reading comprehension – the modality tested by most previous studies. The results of our review allow for a better understanding of when and how a form-based route is used to retrieve a noun’s gender as a function of task requirements and the degree of gender transparency in a language. Our second goal is to extend the proposal of a dual mechanism of gender retrieval by integrating it within a general model of lexical access in comprehension. Current models of lexical access and gender were designed for language production and did not consider the role of word-form in gender retrieval (WEAVER++ by Levelt et al., 1999; Independent Network Model by Caramazza, 1997). We bridge this gap by incorporating a form-based gender access route to an existing model of lexical access in language comprehension: the AUSTRAL (Activation Using Structurally Tiered Representations and Lemmas) model (Taft, 2003, 2006, 2015, 2023). The AUSTRAL model has many similarities to the WEAVER++ model of language production, but it has a bottom-up rather than a top-down information flow. We posit the existence of activation-based gender nodes connected to an abstract lemma level and discuss how the use of a dual route might differ depending on the type of noun (regular, ambiguous, irregular), the degree of gender transparency, and the type of task.

## Literature on gender transparency and lexical access in comprehension

### Systematic search of the literature

**Fig. 1:**
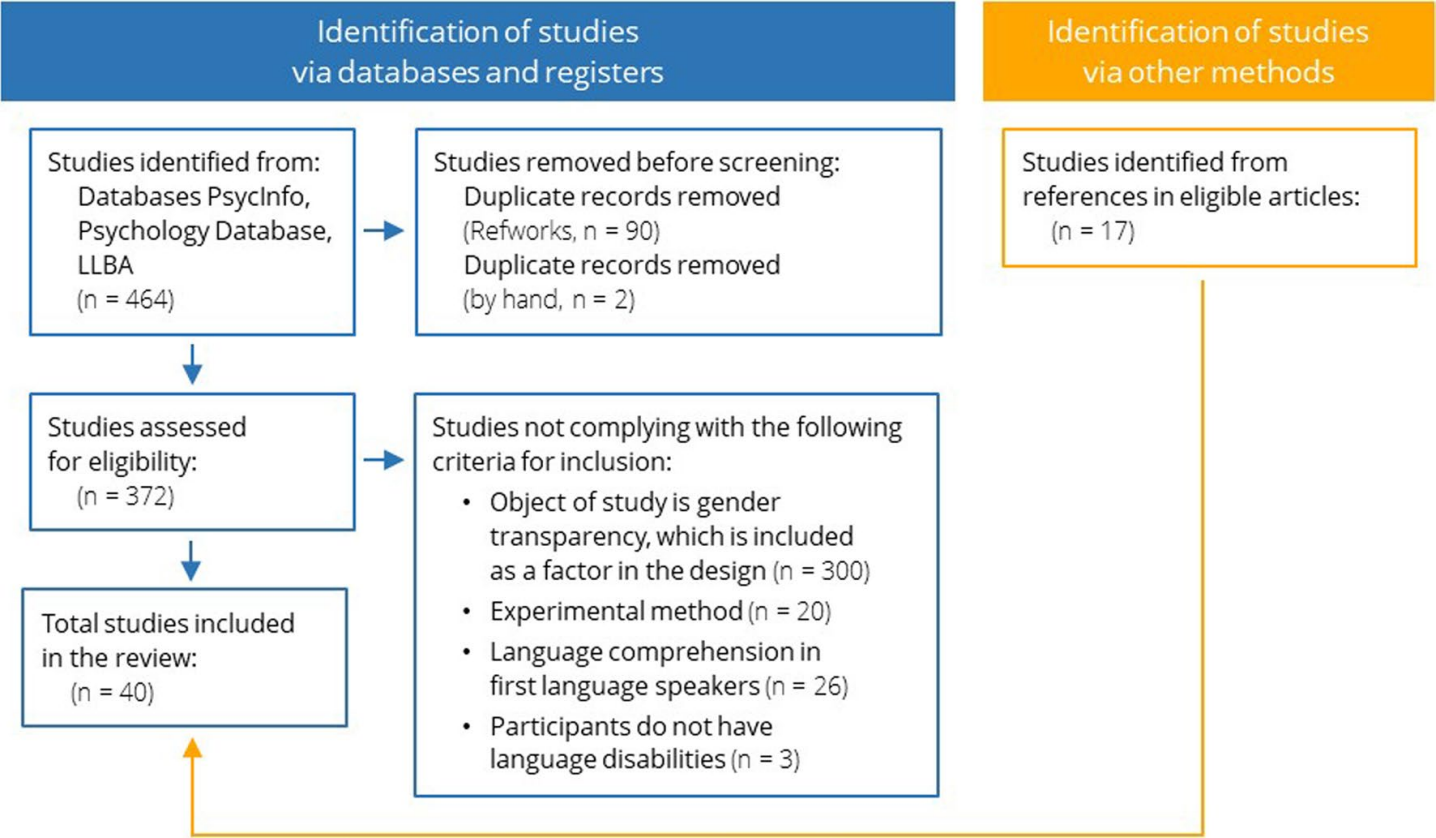
Preferred Reporting Items for Systematic Reviews and Meta-Analyses (PRISMA) diagram of the systematic search. *Note.* According to the updated PRISMA (Page et al., 2021)

We conducted two systematic searches with the keywords “grammatical gender” combined with keywords “transparency” and “regularity,” respectively, in the databases PsycInfo, Psychology Database, and Linguistics and Language Behaviour Abstracts (LLBA). Searches were filtered for scientific journals and for peer-reviewed articles. We followed the Preferred Reporting Items for Systematic Reviews and Meta-Analyses (PRISMA) guidelines for the process of our systematic search (Fig. 1), which yielded 464 results. After removing duplicates with the software RefWorks ® (n = 90) and by hand (n = 2), a total of 372 studies remained. These studies were filtered according to the criteria summarized in Fig. 1.

The application of the criteria above yielded 23 studies (Afonso et al., 2013; de Resende et al., 2019; Aguirre, 2016; Ayoun, 2018; Bates et al., 1995; Bobb et al., 2015; Caffarra & Barber, 2015; Caffarra et al., 2014; Colé et al., 2003; De Martino et al., 2017; Deutsch et al., 1999; Garrido-Pozú, 2022; Gollan & Frost, 2001; Hernández et al., 2007; Hohlfeld, 2006; Holmes & Bâtie, 1999; Holmes & Segui, 2004, 2006; Padovani & Cacciari, 2003; Padovani et al., 2005; Quiñones et al., 2018; Schiller et al., 2003; Spalek et al., 2008). A search of their reference lists allowed us to obtain 17 new studies not found in the initial search (Andonova et al., 2004; Bates et al., 1996; Caffarra et al., 2015, 2017; De Martino et al., 2011; Desrochers et al., 1989; Desrochers & Paivio, 1990; Heim et al., 2005; Hernández et al., 2004; Köpcke & Zubin, 1983; Muller-Gass et al., 2000; Russo et al., 2021; Sekerina et al., 2006; Schwichtenberg & Schiller, 2004; Taft & Meunier, 1998; Tucker et al., 1968; Urrutia et al., 2009). A total of 40 studies were kept in the final sample (see link in *Data availability* section).

### Analysis of the literature

We organized the description of the literature depending on the type of task and the degree of gender transparency of the language under study. The tasks are categorized following a continuum based on how explicit the use of gender is for an experimental participant (Table 1). Such a distinction is important, because explicit methods might encourage participants to use a form-based route in order to better perform in the task. Due to this, results from explicit tasks might not necessarily speak to the retrieval of gender in everyday language comprehension.

**Table 1:** Summary of the tasks used in the reviewed studies

Task	Task type	Task description	Measurements
Gender categorization task	Explicit	Participants press keys depending on noun gender. Categorization tasks can have a time limit, or not – when they do not, rather than pressing keys, participants may be required to write or saying the gender aloud	Accuracy and response times
Grammaticality judgment task	Intermediate explicitness	Participants press different keys (correct/incorrect) depending on the presence of grammatical errors in phrases or sentences	Accuracy and response times
Sentence reading task with embedded gender errors	Intermediate explicitness	Participants silently read sentences that include grammatical errors (e.g., in gender agreement)	Eye-tracking and electrophysiological measures, such as mean component amplitude and latency, or number of fixations and regressions
Marked gender-form prediction task	Intermediate explicitness	Participants read or listen to sentences and are asked to choose the best completion (e.g., a choice between the masculine and feminine form of an adjective in Spanish)	Response times and eye-tracking measures
Inflectional task	Intermediate explicitness	Participants are presented with the singular or the plural form of a noun and must produce its counterpart. It is used with languages in which the number inflection of the noun depends on its gender	Accuracy and response times
Visual world eye-tracking	Implicit	Participants select between multiple pictures based on spoken sentences. Some of these pictures represent nouns that overlap in some critical dimension with the target noun in the sentence – e.g., they might be more or less transparent than the target noun	Response times and eye-tracking measures
Lexical decision task	Implicit	Participants must decide whether a string of letters corresponds to an existing noun	Accuracy and response times
Word naming task	Implicit	Participants read aloud written words. In the sentential variation of this task, words are presented within sentences	Response times
Word repetition task	Implicit	Participants must read aloud the second word in a pair. The pair is manipulated to align in some dimension, e.g., gender congruency	Response times

The degree of explicitness of grammaticality judgment tasks and sentence reading tasks depends on several factors, e.g., whether grammatical errors only involve gender. Inflectional tasks, word-naming tasks, and word-repetition tasks span comprehension and production modalities

On the explicit side of the continuum in Table 1 are gender-categorization tasks, in which participants are presented with nouns and have to press different keys depending on the gender value of each noun. On the implicit side are tasks in which participants are not instructed to attend to gender. An example is a lexical decision task (LDT), in which participants press keys to decide whether a string of letters forms a real word. Along the continuum there are other procedures that show variation in the degree of metalinguistic knowledge required from participants. For instance, in grammaticality judgment tasks, participants are presented with sentences with agreement errors and must decide whether the sentences are grammatically correct or not. Overt gender classification is not required in these tasks, but participants may develop metalinguistic processes of gender monitorization that affect how gender is retrieved. Tasks that cannot be considered fully explicit or fully implicit are referred to as “intermediate.”

Within each task-related subsection (explicit, intermediate, and implicit), we analyze the results according to the degree of gender transparency of languages. We start by describing results with languages that are mostly gender transparent (e.g., Italian and Spanish), and then move through the continuum of gender transparency (e.g., Bulgarian, Hebrew, Russian) towards languages located near the opaque end of the continuum (e.g., French and German).

#### Studies with explicit tasks

The descriptions below focus on comparing the processing of regular, irregular, and ambiguous nouns. Such comparisons are key to assessing the involvement of lexical versus form-based routes in gender assignment. Specifically, if nouns are categorized through a form-based route, then form regularities should affect the retrieval of gender with regular nouns. Meanwhile, a lexical route should be mostly engaged in the retrieval of ambiguous nouns, with potentially less of a role in regular nouns. However, if the use of a form-based route is also affected by the explicitness of a task, such that participants use a form-based route more often in tasks that require explicit categorization, then irregular nouns should be especially tricky. Specifically, regular nouns might hold an advantage over ambiguous and irregular nouns, and this advantage should be reflected, for example, in lower response times and higher accuracy rates. By contrast, irregular nouns should display the highest response times and the lowest accuracy rates. This is because a participant might prioritize the form-based route for the sake of the task, while diminishing the role of a lexical route. However, when an irregular noun is encountered, for a correct response to be given, the participant has to inhibit the form-based route. Inhibiting a route that is being prioritized due to the nature of the task can be especially hard, resulting in irregular nouns triggering the worst performance in the task. Thus, whilst regular nouns should hold the lowest response times and highest accuracy rates, ambiguous nouns should have higher response times and lower accuracy rates than regular nouns, but lower response times and higher accuracy rates than irregular nouns.

Importantly, relying on the form-based route might also depend on the transparency of a language, such that the higher the degree of transparency, the higher the probability that participants rely on a form-based route.

##### Gender transparent languages: Italian and Spanish

In categorization tasks in Spanish and Italian, gender regularities – the “-a” and “-o” cues – have been shown to be crucial, such that regular nouns consistently elicit higher accuracy and lower response times in Italian (Bates et al., 1995, 1996; Padovani & Cacciari, 2003, Padovani et al., 2005) and Spanish (Afonso et al., 2013; Aguirre, 2016; Caffarra et al., 2017; Hernández et al., 2004). Meanwhile, ambiguous and irregular nouns seem to be categorized through the retrieval of definite articles (Muller-Gass et al., 2000).

Interestingly, only Afonso et al. (2013) considered gender regularities that are not “-o” and “-a”, i.e., suffixes and pseudosuffixes that are less common but strongly correlated with gender (e.g., the feminine-marking endings “-ción” or “-zione” in Spanish and Italian). Afonso and colleagues (2013) tested the categorization of regular and irregular Spanish nouns. The results revealed an advantage of regular over irregular nouns – shorter response times and higher accuracy rates – only for nouns that ended in “-o” and “-a” but not for nouns featuring other regularities. In Italian, Padovani & Cacciari, 2003, Padovani et al., 2005) compared the categorization of ambiguous and irregular nouns and found that the latter elicited the longest latencies (Padovani & Cacciari, 2003, 2005).

In some of these studies, participants reported silently producing the article of ambiguous nouns to make a gender decision, or benefited from the presence of an agreeing article presented as a prime only in the case of ambiguous or irregular nouns (Afonso et al., 2013; Hernández et al., 2004). In line with this, in categorization tasks that used functional magnetic resonance imaging (fMRI) measurements, Hernández et al. (2007) and Padovani et al. (2005) found increased activation in areas related to the phonological processing of determiners before ambiguous and irregular nouns (increased activation in BA 44, BA 55, and the bilateral insula near BA 47; Heim et al., 2002; Miceli et al., 2002).

To summarize, gender-categorization tasks in Italian and Spanish support the use of both a lexical and a form-based route to retrieve gender, which confers a processing advantage to regular nouns. When confronted with irregular and ambiguous nouns, participants seem to rely on the silent production of determiners. This suggests that the sole use of a lexical route for ambiguous nouns – in which gender is retrieved from the noun’s lexical entry – and the conflict between the form-based and lexical route in irregular nouns triggers a processing difficulty when categorizing them according to gender.

##### Intermediate gender-transparent languages: Bulgarian, Hebrew

Only two studies, on Bulgarian and Hebrew, conducted gender-categorization tasks in languages without simple “-o/-a” cues like Spanish and Italian, but still more gender-transparent than French and German. Both studies found an advantage of regular over ambiguous and irregular nouns, consistent with the hypothesis that participants recovered gender through a form-based route.

In Bulgarian, most masculine nouns end in a consonant, feminine nouns end in “-a/-ia”, and neuter nouns end in other vowels. The feminine gender is considered the most transparent gender value because it is the most easily predicted from the noun form. In a categorization task, Andonova and colleagues (2004) found lower response times and higher accuracy rates for regular than irregular nouns, with feminine nouns showing an advantage over the other genders.

Meanwhile, Hebrew has only two gender values, masculine and feminine. Three different phonemes mark the feminine value of inanimate nouns: /ah/, /et/, and /it/, while any other endings are associated with masculine gender. As in Bulgarian, the feminine can be considered the most transparent gender value. In a gender-categorization task, Gollan & Frost (2001) found that regular feminine and masculine nouns were categorized faster and more accurately than irregular nouns (i.e., feminine nouns that lacked the three endings above). Importantly, regular feminine nouns elicited lower response times than masculine regular nouns. In a second experiment, the effect of regularity disappeared in response times when the regular feminine nouns were removed, such that the gender cues were all masculine endings. These results suggest that Hebrew speakers use form regularities to categorize nouns, and that categorization is easier the fewer the regularities associated with a gender value – three for the feminine versus all other endings for the masculine.

Overall, these results support the existence of a form-based route operating in the presence of gender regularities. The results also suggest that the greater the number of endings associated with a gender value (i.e., and hence the lower the transparency of a gender value; Kupisch et al., 2018), the lower the involvement of a form-based route. The study of Gollan & Frost (2001) also suggests that when transparency is reduced, a form-based route might become insufficient to give a quick and reliable response, such that gender retrieval occurs similarly for regular and irregular nouns. In such cases, gender retrieval might occur mainly through a lexical route.

##### Languages towards the opaque end of the transparency continuum: French, German

Categorization results with less transparent languages like French and German are consistent with results from Bulgarian and Hebrew, in that they suggest that the lower the degree of transparency, the lesser the role of the form-based route. For example, French speakers seem to benefit from the presence of gender regularities: they show lower response times and higher accuracy rates when deciding the gender of regular versus ambiguous nouns (Desrochers et al., 1989; Desrochers & Paivio, 1990; Holmes & de la Bâtie, 1999; Holmes & Segui, 2004, 2006; Taft & Meunier, 1998; Tucker et al., 1968). Similar to speakers of intermediately transparent languages, French speakers seem to use a strategy of evoking articles to categorize nouns, but they do so even with regular nouns.

Several types of evidence support the generalization above. First, the advantage of regular nouns disappears for nouns that begin with a vowel, for which the preceding definite article is invariable across genders (Ayoun, 2018; Desrochers & Brabant, 1995; Desrochers & Paivio, 1990; Desrochers et al., 1989; Taft & Meunier, 1998). For instance, in Experiment 2 of Taft & Meunier (1998; not included in the final sample of the review due to “gender transparency” not being a factor in their design), gender decisions for regular nouns showed increased response times when the article that participants could have generated did not help identify the noun’s gender (e.g., place names beginning with a vowel, for which the only possible article is “*l*”). In a previous experiment (Experiment 1), the researchers also demonstrated that gender classification times were longer for irregular than regular words regardless of their word frequency. Given that frequency is a diagnostic of lexical access, this result was taken to imply that lexical gender information was primarily used, but that form-based information also played a role. A key role of article information is also supported by findings in Ayoun (2018). Participants in this study reported using definite articles to decide the gender of the target noun, regardless of whether a gender regularity was present. Moreover, responses were faster when participants were asked to choose between the masculine and feminine indefinite articles than when they were asked to choose between masculine and feminine gender labels (Desrochers et al., 1989).

Findings with German children and adults show that they can rely on gender regularities, especially when asked to assign gender in untimed tasks to pseudowords displaying those regularities (Hohlfeld, 2006; Köpcke & Zubin, 1983; Mills, 1986; Schwichtenberg & Schiller, 2004; Szagun et al., 2007). Yet, in an online gender-categorization task, Hohlfeld (2006) failed to find an effect of gender transparency: regular nouns did not show lower response times or higher accuracy than ambiguous nouns. However, their experimental setup might have been underpowered to detect potential effects due to comprising only 16 participants. Meanwhile, Bobb and colleagues (2015) did find a response time advantage for regular nouns, but their regular nouns only included feminine nouns ending in “-e” (the simplest gender cue in German), and did not consider other regularities. This may have encouraged participants to create a task-based heuristic applied to nouns ending in “-e”. Importantly, evidence from an fMRI study with a categorization task suggests that German speakers also use determiners to decide the gender of the word (Heim et al., 2005). This was supported by reports from the participants themselves, as well as increased activity in the brain area BA 45, which is related to the phonological processing of determiners (Miceli et al., 2002). Unfortunately, this study used regular and ambiguous nouns interchangeably, and thus they were not analyzed based on gender transparency.

An interesting study in German is that of Schiller et al. (2003). The researchers conducted a gender-categorization task within a go/no-go paradigm in which participants had to select a masculine or feminine definite article for each noun. In the go/no-go paradigm participants have to give a response under a specific condition (the “go-signal”), and avoid a response under another condition. In Schiller et al. (2003), go/no-go conditions were counterbalanced across two blocks and could hence be restricted to either masculine or feminine nouns. Critically, the nouns could be regular or ambiguous, but they could also have a natural/biological gender, such that their gender cues were not only formal but also semantic. Response times and N200 brain responses were recorded. The N200 is a negative deflection of the event-related potential (ERP) response related to response inhibition and typically elicited in go/no-go paradigms. The peak of the N200 appears in no-go conditions when a minimum amount of information for giving a response is available. Thus, the peak for the N200 should appear earlier for regular nouns in comparison to ambiguous nouns, and for nouns with natural gender in comparison to nouns with grammatical gender.

The results showed that regular nouns elicited lower response times than ambiguous nouns, but no differences in the N200 component latency. By contrast, the presence/absence of semantic cues evoked differences in response times and also in the latency of the peak of the N200. From the perspective of a form-based route, the lack of an effect in response times might suggest that gender regularities by themselves were insufficient for participants to perform the go/no-go task. A possible explanation is that gender cues in German are not part of lexical access, but rather of a post-lexical verification phase in which participants self-monitor the correctness of the article chosen for categorization. Response times would be more sensitive to these late post-lexical effects, while electrophysiological components might be more sensitive to early effects.

##### Summary of results with explicit tasks

Even though the lexical route is the main route of gender retrieval, our review of gender categorization studies supports the use of a form-based route in languages at different points of the transparency continuum. A form-based route seems to be highly active in transparent languages with regular “-o/-a” nominal endings, but less active in less transparent languages like French and German, or in nouns with inconsistent form-to-gender mappings – i.e., with more than one gender regularity per gender value. In such cases, participants likely rely more on a lexical route and/or on the silent production of determiners. Further, irregular nouns uniformly elicit processing difficulties across languages. This suggests that participants prioritize a form-based over a lexical route in categorization tasks and have difficulties inhibiting form cues when they are misleading – as with irregular nouns. The evidence suggests that irregular nouns are also categorized through the silent production of determiners.

#### Studies using intermediately explicit tasks

In intermediately explicit tasks, participants’ attention is not directly focused on gender, and hence a form-based route might not be the main mechanism used in the task. Hence, participants might rely both on lexical and form-based routes to retrieve the gender of regular nouns. Caffarra and colleagues (2014) proposed that the use of the two routes may require additional cognitive resources in comparison to ambiguous nouns, especially if transparent nouns have to be decomposed and if the output of both routes needs to be verified, a matching process that might increase processing time and resources. Thus, processing measures – like response times and brain responses – might increase for regular nouns, which should reduce the processing advantage previously described for regular nouns versus ambiguous nouns in explicit tasks. Meanwhile, irregular nouns should continue to be cognitively demanding, given the conflict in the outputs of the lexical and form-based routes, which should trigger extra verification processes. Results with tasks of intermediate explicitness are summarized below. Note that there is no section dedicated to French and German because, to our knowledge, no studies complying with our inclusion criteria have been conducted in these languages.

##### Gender-transparent languages: Italian and Spanish

The available evidence in transparent languages seems to support the use of a dual route to access the gender of regular nouns. Several studies find that, in comparison to ambiguous and irregular nouns, regular nouns trigger higher brain responses, which tend to emerge early – between 200 and 400 ms – and are maintained until later time windows, consistent with increased processing effort (Caffarra & Barber, 2015; Caffarra et al., 2014, 2015, 2017). For example, in several judgment tasks with gender-agreement errors in Italian, Caffarra and colleagues reported early effects of transparency in the left hemisphere, with regular nouns showing greater amplitudes across multiple components compared to ambiguous and irregular nouns (Caffarra et al., 2014, 2015, 2017; Caffarra & Barber, 2015).

The studies of Cafarra and colleagues manipulated two factors: agreement between nouns and words like articles (agreement vs. disagreement), and the transparency of the nouns (transparent vs. opaque/irregular, depending on the study). Interestingly, most results did not support an interaction between transparency and agreement: increased brain responses for regular nouns were observed regardless of whether these nouns appeared in the context of agreeing or disagreeing determiners. An exception is Caffarra et al. (2014), who observed that the disagreeing condition was significantly more negative than the agreeing condition for regular nouns after 700 ms. The authors interpreted this result as a post-lexical task-related effect, perhaps reflecting a verification of the matching between form-cues and the agreement element, which “failed” in the disagreeing condition. The reason why such an interaction between transparency and agreement was not observed in other studies may be related to the increased explicitness of the method used by Caffarra et al. (2014). In contrast with other studies, they included exclusively gender errors and short noun phrases, rather than longer sentences. This could have boosted participants’ reliance on the form-based route to successfully detect agreement errors.

The suggestion above is consistent with the results of a grammaticality judgment task with fMRI featuring Spanish noun phrases with or without gender agreement errors (Quiñones et al., 2018). The results showed increased activity for regular nouns in the left fronto-temporal, parietal, and occipito-temporal regions plus the supramarginal gyrus in both the agreement and disagreement conditions. It was suggested that activation in these areas reflected processes of a lexical nature but also of morphological decomposition with regular nouns, along with an associated cost due to the decoding of redundant morphological information in the agreeing condition. Conversely, ambiguous nouns showed an increase of activation on the supramarginal and angular gyri, possibly associated with a processing cost in the integration of the morphosyntactic information in the disagreement condition.

While the brain-related results of the studies above are consistent, the behavioral results are in conflict. Specifically, Caffara et al. (2014) found an effect of gender transparency in accuracy (fewer errors for regular than ambiguous nouns), while Quiñones et al. (2018) observed the opposite pattern: more errors for regular than ambiguous nouns, and an interaction in response times between gender transparency and agreement. This interaction showed that latency differences between agreement and disagreement conditions were greater for ambiguous nouns. The reasons for the conflicting results across studies are unclear, and more empirical studies are needed.

Finally, the study of Garrido-Pozú (2022) used a paradigm in which gender regularities were included as a factor for the prediction of adjectives in Spanish (e.g., "perfecto" vs. "perfecta" (masculine and feminine form for adjective “perfect”). The author used eye movements to measure participants’ predictive skills. Participants saw two written adjective forms that ended sentences presented auditorily. The last three words of these sentences consisted of a determiner that did or did not mark agreement (gendered: definite article [“el” _masculine_ vs. “la” _feminine_]/ ungendered: possessive determiner “his/her/their” [“su” _masculine/feminine_]), a noun that varied in gender transparency (regular [“fiesta” _feminine_, party] or ambiguous [“lección” _feminine_, lesson), and the target adjective. Participants showed faster recognition of the target adjective when it was preceded by a gendered article than when it was preceded by a possessive determiner, but only for sentences with ambiguous nouns. By contrast, the choice between adjectival forms following regular nouns did not show differences depending on the presence of an agreeing article. This study is therefore in line with explicit studies showing that ambiguous nouns benefit from the presence of determiners during categorization tasks (Hernández et al., 2004, 2007), and extends this finding to gender predictions in a more implicit task.

In sum, results from intermediately explicit tasks in transparent languages suggest that retrieving the gender of regular nouns involves both form-based and lexical routes: regular nouns elicit higher brain responses than ambiguous nouns, consistent with additional morphological decomposition processes. By contrast, ambiguous nouns seem to solely involve a lexical route. Finally, and contrary to our initial predictions, irregular nouns show lower ERP amplitudes than regular nouns, which suggests that a form-based route might not be used with irregular nouns.

##### Intermediate gender-transparent languages: Hebrew

The only study with a language of intermediate transparency was conducted in Hebrew and is in line with results from Italian and Spanish. Gollan & Frost (2001) conducted a grammaticality judgment task in Hebrew with nouns following gender-marked adjectives. Irregular feminine nouns and regular masculine nouns were used. The results showed that form regularity did not have an effect when the noun and adjective agreed in gender, but it did play a role when they disagreed: participants were faster and more accurate to detect agreement violations with regular than irregular nouns in the disagreement conditions. This is in line with the results of Cafarra et al. (2014) and Quiñones et al. (2018), who found interactions between noun form regularity and agreement in the most explicit version of the grammaticality judgment task. Thus, the results in Hebrew suggest that gender regularities are used to detect agreement errors, consistent with the use of a form-based route for gender retrieval.

##### Summary of results with intermediately explicit tasks

Our review of intermediately explicit tasks and especially with paradigms involving brain responses suggests that in transparent languages, gender retrieval with regular nouns is costlier than with ambiguous or irregular nouns. This supports the joint use of lexical and form-based routes. Further, evidence from transparent and intermediately transparent languages suggests that comprehenders rely on a form-based route to detect agreement errors if a task is more explicit with regard to gender – for example, if a judgment task features only gender errors. In transparent languages, a form-based route seems to be used to make gender predictions.

#### Studies with implicit tasks

In implicit tasks, participants are presumably unaware that gender is the object of study and thus they might deploy both lexical and form-based routes to retrieve the gender of regular nouns – as opposed to strategically relying on a form-based route as in explicit tasks. Assuming that the use of lexical and form-based routes with regular nouns is cognitively more demanding than the sole use of a lexical route in ambiguous nouns, regular nouns should elicit more processing cost than ambiguous nouns. This predicts that response times in tasks like word naming or lexical decision should be higher for regular than ambiguous nouns. Finally, while irregular nouns are predicted to be particularly costly, recall from the previous section that Caffarra et al. (2015) found more processing cost for regular than irregular nouns, which raises the possibility that a form-based route might not be used with irregular nouns.

##### Gender transparent languages: Italian and Spanish

One must be cautious when drawing conclusions about some of the studies presented here, since they may suffer from insufficient statistical power: Results are often conflicting, marginally significant, or significant by participants but not by items, suggesting a need for replication (Brysbaert, 2019; Vasishth & Gelman, 2021). But to the extent that they can be trusted, these results point to the involvement of a form-based route with regular nouns.

We first discuss LDT and then word-naming tasks, since their outcome is slightly different. In a LDT in Spanish, Urrutia et al. (2009: Experiment 2) found an effect of regularity only by participants: regular nouns displayed higher response times than ambiguous nouns. In an LDT in Italian, De Martino et al (2011: Experiment 5) found that irregular nouns elicited more errors than regular nouns, without any response time differences. But in a later study, differences between regular and irregular nouns were only significant by participants for accuracy, and irregular nouns showed higher response times than regular nouns (De Martino et al., 2017). Meanwhile, in word-naming tasks in Italian, regular nouns showed higher response times and fewer errors than irregular nouns (De Martino et al., 2011, 2017). Yet, in the case of response times, differences only arose by participants (De Martino et al., 2011) or were significant by participants and marginally significant by items (De Martino et al., 2017). Importantly, no differences have been reported when, instead of irregular nouns, ambiguous and regular nouns were compared (Bates et al., 1995).

Summarizing these findings: regular nouns tend to elicit higher response times than ambiguous nouns in LDTs in Spanish, and than irregular nouns in word-naming tasks in Italian. This goes in line with the hypothesis that the retrieval of gender with regular nouns relies on two routes and thus is cognitively more demanding. Yet, these results also suggest that irregular nouns are processed more quickly than regular nouns, in line with findings from Caffarra et al. (2015). The fact that irregular nouns elicited more errors and seemingly higher response times than regular nouns in LDTs (i.e., that it was harder to identify an irregular than a regular noun), might be related to how infrequent it is for a noun that has a form ending in "-o" and "-a" to be feminine and masculine, respectively. Indeed, the consistency of ortho-phonological patterns is known to influence the LDT performance (Grainger & Jacobs, 1996; Peereman et al., 2009). Since this ortho-phonological and grammatical pattern is less frequent, when the specific aim of the task is to identify existing or non-existing words, irregular nouns may experience a disadvantage compared to regular nouns.

The study of De Martino et al. (2011) provides further insight into the mechanisms used for retrieving gender in irregular nouns. The authors also conducted several singular-plural inflectional tasks in Italian. In one of their experiments, participants were asked to produce the singular form of a visually presented plural noun. The plural forms were regular (e.g., “diplomi”_;_ “-i” is the regular plural ending for masculine nouns), but the singular forms were irregular (the singular form of “diplomi” is “diploma”, which ends with “-a”, a form typically associated with feminine nouns). The rationale was as follows: if a form-based route was used to retrieve the gender of singular nouns, the production of nouns with irregular singulars should be delayed in comparison with nouns whose plural and singular forms are both regular. This is exactly what was found. In another experiment, De Martino et al. (2011) presented irregular singular forms like “diploma” and asked participants to produce their (regular) plural forms. According to the logic above, the production of a plural form should not show differences in comparison to fully regular nouns because the irregularity should be solved during the reading of the word, such that the selected gender value should activate consistent information between gender and number values. Again, this prediction was met.

Interestingly, Russo et al. (2021), in a similar inflectional task in Italian with fMRI, observed increased activity on the cerebellum and the right middle temporal gyrus (MTG) for the conditions involving irregular nouns versus only regular and/or ambiguous nouns. The higher activation of the cerebellum has been taken to reflect greater executive attentional control, whilst the higher activation of the right MTG has been associated with inhibitory mechanisms (Marangolo et al., 2003, 2006).

Taken together, these results suggest that the form-based and lexical routes are used to retrieve the gender of regular nouns, but that the form-based route is inhibited with irregular nouns, which reduces processing cost.

##### Intermediate gender-transparent languages: Hebrew, Russian

Results from implicit tasks in these languages are inconsistent, but the reason might be related to the different dependent measures used across tasks. Deutsch et al. (1999) conducted a word-naming task in Hebrew in which participants read aloud sentences (sentential primes with either regular or irregular nouns) followed by a target verb that always agreed in gender with the noun. Naming times showed no differences in the naming of the agreeing verb depending on the regularity of the previous noun. This is in line with Gollan & Frost (2001), who proposed that in the absence of agreement errors, a form-based route is not used in Hebrew. Yet, in the case of Spanish and Italian, much of the evidence supporting a form-based route was obtained through measures that were not response times, mainly EEGs and fMRI.

A study in Russian using an implicit task and a more fine-grained measure did find evidence supporting a form-based route (Sekerina et al., 2006). Russian is a language that has a degree of gender transparency that could be considered similar to that of Hebrew (Corbett, 1991). Nouns ending in non-palatal consonants are masculine, nouns ending in a stressed [a] are mostly feminine, and nouns ending in a stressed [o] are neuter (Mitrofanova et al., 2018), with the other endings being ambiguous. Sekerina et al. (2006) conducted a visual world eye-tracking paradigm in which participants heard nouns preceded by adjectives that agreed with a target noun and were asked to click on the noun mentioned in the instruction (e.g., “Look at the blue…”). The target noun was depicted within a set of pictures, and its gender value differed from that of a competitor noun – also pictured. The target and competitor noun were either regular or ambiguous. The results showed that the percentage of fixations to the competitor was higher when both nouns were ambiguous in comparison to when they were both transparent. Thus, it was concluded that predictive agreement was more difficult to establish when nouns are ambiguous rather than regular. This result supports the hypothesis that a form-based route is used with regular nouns even in languages not as transparent as Italian and Spanish.

##### Languages towards the opaque end of the transparency continuum: French, German

Implicit tasks manipulating gender transparency have not been conducted in German. In French, significant effects related to gender transparency in lexical decision and word-naming tasks have been reported in one study but only with low-frequency words (Colé et al., 2003). Other studies have failed to obtain them (Desrochers et al., 1989; Desrochers & Paivio, 1990; Spalek et al., 2008). More specifically, the LDT of Colé et al. (2003) showed that regular nouns had lower response times than ambiguous and irregular nouns, yet ambiguous and irregular nouns were intermixed together (“nouns with low-predictiveness endings”). It is unclear how to interpret these results because they go against lexical decision results in Spanish and hence against the logic behind a double route of gender retrieval with regular nouns. A possible explanation concerns the mixing of ambiguous and irregular nouns in the same condition. As previously shown and discussed, irregular nouns been found to elicit higher response times than regular nouns in LDTs. However, further studies are necessary.

Colé and colleagues (2003) also used eye-tracking techniques in an LDT with article-noun pairs that always agreed in gender. Their results supported the involvement of a form-based route with regular nouns. They first discarded participants that displayed a single-fixation strategy – who only looked once to the noun phrase – and then analyzed those participants who made a second fixation before responding. The results showed that the probability of observing a second saccade on a preceding article for feminine nouns was higher with ambiguous than regular nouns. Further, when the second saccade on the noun phrase was directed towards the noun, the probability of the following fixation to occur on the noun’s ending was higher for regular nouns than for ambiguous nouns. These results suggest that gender regularities influence implicit reading profiles and that agreeing articles are important for ambiguous nouns during agreement.

##### Summary of the results with implicit tasks

Studies with implicit tasks are currently scarce and often display inconsistent results. To the extent that these studies can be interpreted, in transparent languages, they suggest that a form-based route is used with regular nouns but inhibited with irregular nouns. Studies on languages with lower degrees of gender transparency – from Russian to French – further suggest that a form-based route is used to predict the gender of upcoming words and/or to compute agreement. Lastly, the evidence suggests that effects of gender transparency might be harder to capture with response times in lexical decision and naming tasks than with other more fine-grained measures (e.g., eye-tracking, fMRI). However, the conflicting findings with response-time studies might also be due to insufficiently powered designs.

## Discussion

We reviewed experimental studies to determine the role of noun-form transparency in gender processing. Our goal was to study the involvement of a dual-route mechanism across languages. Our review considered the task used in each study – ranging from explicit to implicit tasks – as well as the gender transparency of the languages under study. Table 2 summarizes the results of the review in connection to the dual mechanism of gender selection advocated below.

**Table 2:** Use of the two routes of gender selection according to task explicitness, noun-form transparency, and degree of gender transparency of a language

Use of gender within the task	Noun transparency	Degree of transparency of the language		
		High	Intermediate	Low
**Explicit**	Regular	Primary route: Form-based route Secondary route: Lexical route	Primary route: Form-based route Secondary route: Lexical route	Primary route: Lexical route plus articles Secondary route: Form-based route
	Ambiguous	Lexical route plus articles	Lexical route plus articles	Lexical route plus articles
	Irregular	Primary route: Form-based route Secondary route: Lexical route plus articles	Primary route: Form-based route Secondary route: Lexical route plus articles	Primary route: Lexical route plus articles Secondary route: Form-based route
**Intermediately explicit**	Regular	Primary route: Lexical route Secondary route: Form-based route	Primary route: Lexical-based route plus articles Secondary route: Form-based route	*Evidence not available*
	Ambiguous	Lexical route plus articles	*Evidence not available*	*Evidence not available*
	Irregular	Lexical route plus articles	Lexical route plus articles	*Evidence not available*
**Implicit**	Regular	Primary route: Lexical-based route Secondary route: Form-based route	Primary route: Lexical-based route Secondary route: Form-based route	Primary route: Lexical-based route Secondary route: Form-based route
	Ambiguous	Lexical-based route with articles	Lexical-based route with articles	Lexical-based route with articles
	Irregular	Lexical-based route with articles	*Evidence not available*	Lexical-based route with articles

By “primary route" we mean the route mostly used for gender retrieval in a task. Note that results with implicit tasks are particularly inconsistent and seem to depend on the type of measure (e.g., response times vs. eye-tracking)

Our review suggests that a form-based route is used for the assessed languages. This is because results show differences between regular, ambiguous, and irregular nouns in every language, at least under some experimental conditions. The use of a form-based route seems to be affected by (a) the degree of gender transparency of the language: the higher their degree of gender transparency, the more consistent the results supporting the form-based route; (b) the degree of gender transparency of gender values themselves, i.e., how easy it is to predict a specific gender value from a noun’s form: the higher the number of different cues related to a gender value, the harder it seems to predict it (Kupisch et al., 2018, 2022) — for instance, in Hebrew the form-based route would have a more prominent role for the feminine gender, which is associated with three regularities, than for the masculine gender, which is associated with more regularities; (c) the explicitness of the task: more explicit tasks provide the clearest evidence of a form-based route.

The evidence suggests that regular nouns ending in “-o” and “-a” in the most transparent languages rely on form-based and lexical routes to retrieve gender. Meanwhile, ambiguous nouns in all languages seem to rely on a lexical route. Yet, determiners seem crucial for gender retrieval when the lexical route is the primary route for gender retrieval, or when the form-based route is available but the degree of gender transparency of the language – or of the gender value itself – is not high. Overall, regular nouns seem to be costly in cognitive terms, suggesting that they rely on two routes of gender retrieval. However, contrary to our predictions, irregular nouns do not display the processing cost predicted under the involvement of a form-based route. This suggests that, if active, the form-based route is inhibited very early in the lexical access of irregular nouns.

The theoretical implications of these conclusions are as follows. First, models of lexical access should accommodate a double route of gender access, at least for visual language comprehension. Second, the involvement of a form-based route should be allowed to vary across languages. This suggests that lexical access of gender is not implemented uniformly across languages, such that links between different levels of representation in the lexicon may depend on the characteristics of the language. In the next section we advance a proposal of how a dual mechanism of gender retrieval could be implemented within a general model of lexical access in language comprehension.

## A proposal of gender representation and process within the AUSTRAL (Activation Using Structurally Tiered Representations and Lemmas) model of lexical access

The AUSTRAL model by Taft (2003, 2006, 2015, 2023) incorporates the notion of “lemma” to models of reading language comprehension (see also Crepaldi et al., 2010; Diapendaele et al., 2009). The use of lemmas is advantageous because lemmas are used in language production models to represent gender (Levelt et al., 1999; Roelofs, 2018). More specifically, nouns are thought to have an abstract representation at a lemma level that is connected to syntactic features like word class, number, and gender. The AUSTRAL model implements lemmas that capture correlations between word-form and grammatical features. This makes AUSTRAL a suitable architecture to model gender regularities.

### Locating gender in the AUSTRAL: Gender nodes at a syntactic level

**Fig. 2:**
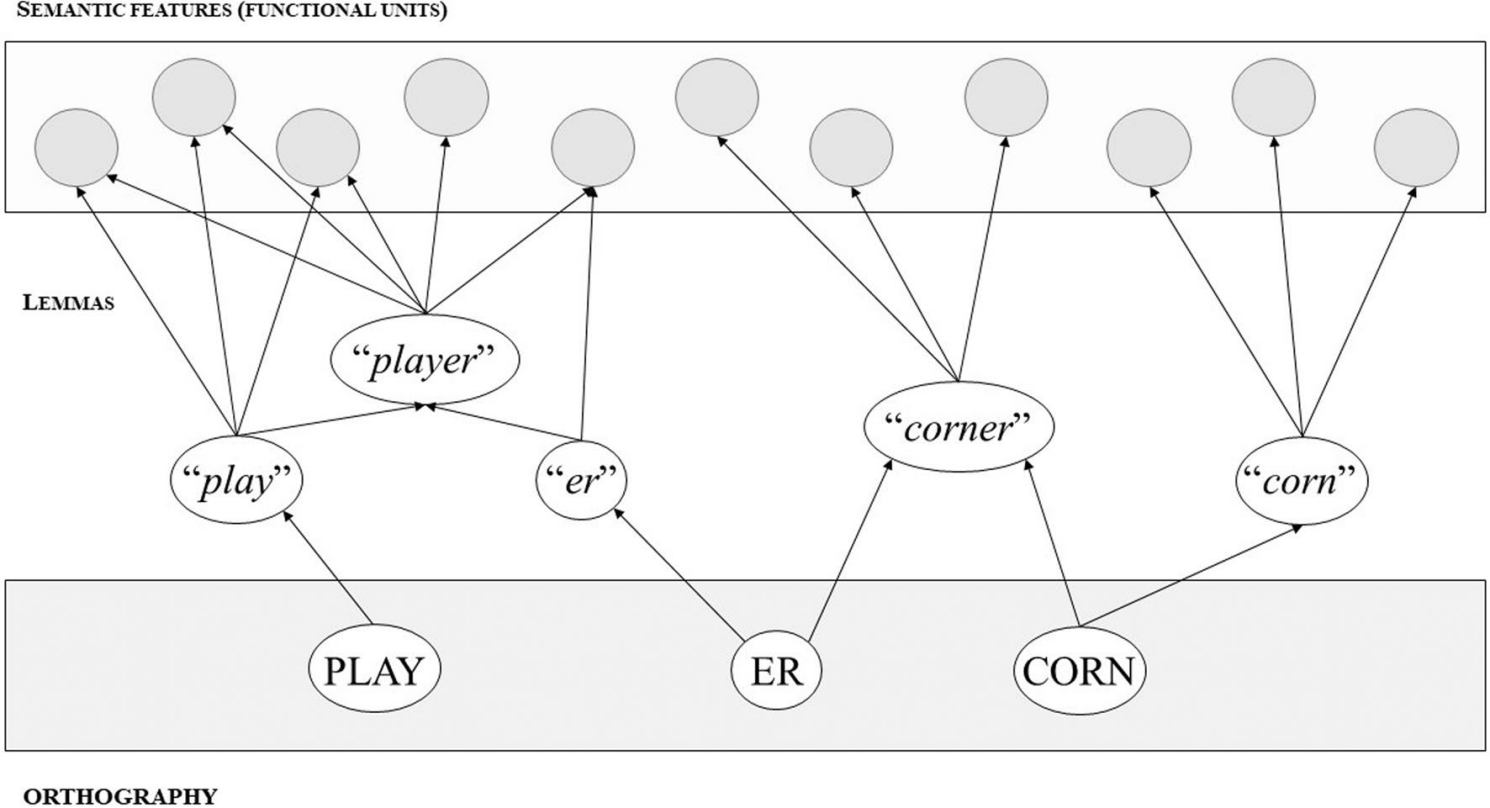
Architecture of the AUSTRAL (Activation Using Structurally Tiered Representations and Lemmas) model. Figure adapted from Taft (2023)

The AUSTRAL model defines three levels of representation that contain the necessary information for word recognition: the word-form level, the lemma level, and the semantic level (Taft, 2023). The lemma level can be defined as a level of representation in which sub-patterns of activation are established: (a) with form-based units at the word-form level; (b) with functional-based units at the semantic level. A lemma is sensitive to (pseudo)morphological decomposition. A word like “player” is decomposed at the word-form level into a stem PLAY- and a morpheme -ER (Fig. 2; note that following AUSTRAL, word forms are represented with uppercase letters and lemmas with quotation marks). These two chunks activate three lemmas: “*play*”, “-*er*”, and, indirectly, “*player.*” As PLAY- occurs across words sharing semantic features (e.g., “play,” “played,” “playtime”), the stem “*play*” is represented as a lemma node that connects the form of every word with these semantic features. Meanwhile, “-*er*” is a suffix that usually refers to someone or something performing an action related to the stem, and hence it is also represented as a lemma. Thus, the lemma “*player*” is selected once a threshold of activation is reached. Importantly, lemmas can be words and affixes, but also pseudo-words like “-vive-”, as in “revive” and “survive” (Taft, 2023).

The AUSTRAL model only includes lemmas that represent associations between word-form units and functional semantically based units. However, these functional units might additionally encode other types of lexical characteristics besides semantics (Taft & Nguyen-Hoan, 2010). Hence, it would be possible to extend the model in order for lemmas to represent form associations with features such as gender. In gendered languages, strings of letters are consistently associated with a gender value, such as the word “screw” always being masculine in Spanish (“tornillo”) but feminine in German (“Schraube”). We follow classic models of language production that represent gender through gender nodes (Levelt et al., 1999). In the context of AUSTRAL, the word-form representation of gendered words would be connected to its respective gender node through the lemma level.

**Fig. 3:**
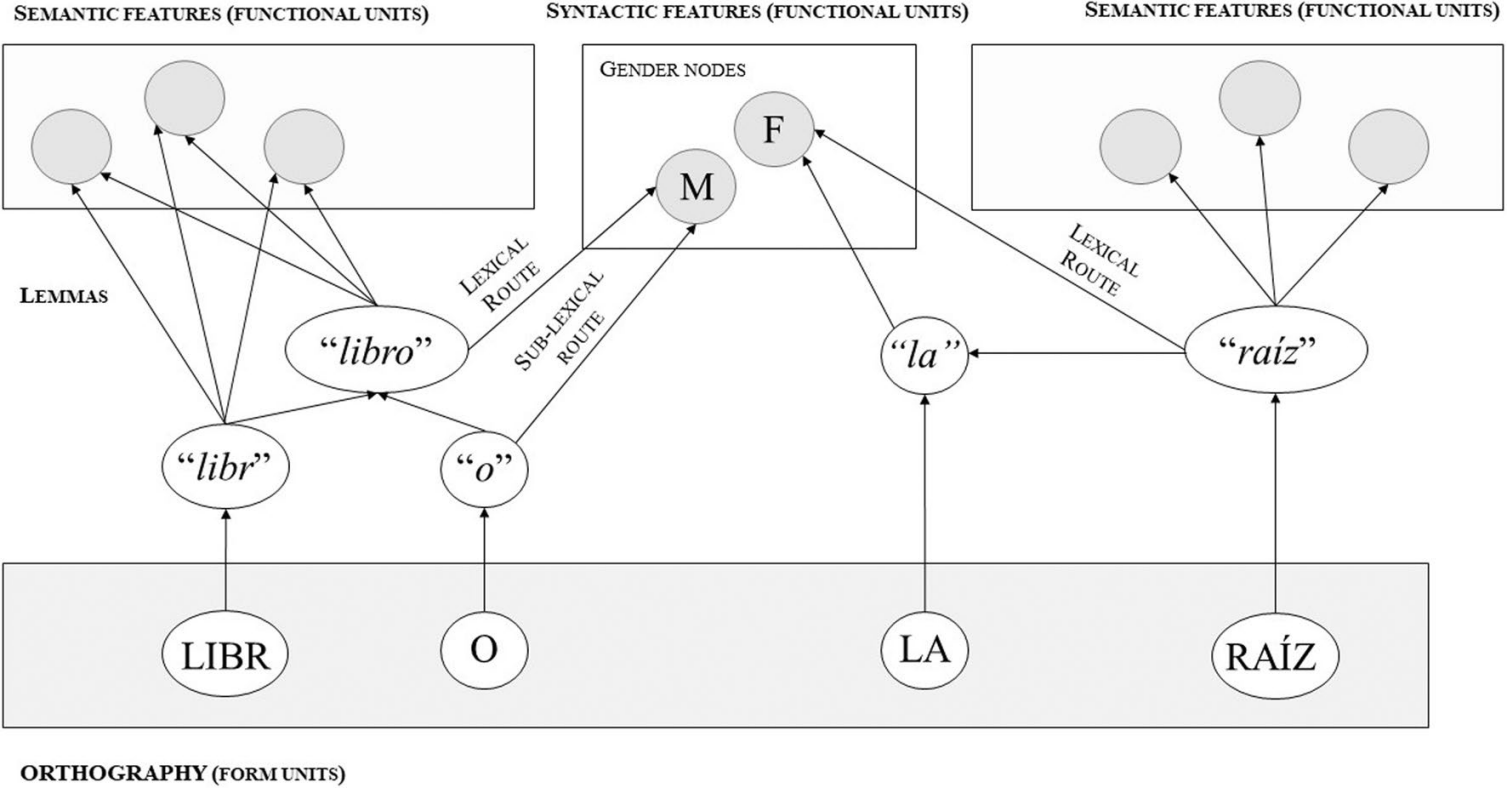
Representation of gender in the modified AUSTRAL model with sub-lexical and lexical routes. *Note*. Retrieval of gender in Spanish during the reading of a regular masculine noun “libro” (book) on the left, and a noun phrase with an ambiguous feminine noun “la raíz” (the root) on the right. Gender retrieval for “libro” occurs through the lexical and sub-lexical routes. Gender retrieval for “raíz” occurs only through a lexical route. Pseudomorphological decomposition takes place for “libro” since “-o” is an ending that correlates with the masculine gender. Nouns have direct connections with their articles (represented in the figure as a definite article). Thus, the definite article “la” activates the feminine gender node, which in turn activates the definite article. F = Feminine, M = Masculine. Adapted from Taft (2023)

A first question is where exactly to place gender nodes within the architecture of AUSTRAL. By understanding the lemma as an intermediate level of correlation between form and function, it does not seem possible for gender nodes to be located at the lemma level. Thus, we have two options: locating gender at the semantic level, or locating it at a separate level of syntactic representation (for a similar idea, see the Independent Network model; Caramazza, 1997). We believe that it is not convenient to locate grammatical gender within the semantic level because recent evidence does not support the claim that gender values always have semantic correlates (Elpers et al., 2022). Thus, we opted to incorporate gender nodes within a separate level of syntactic information. As seen in Fig. 3, activation within the word-form level would spread to the lemma level and in parallel to semantic and syntactic levels of representation.

### Processing gender in regular nouns within AUSTRAL: A dual-route mechanism

One aspect of the AUSTRAL model is key to explain the processing of gender through a dual-route mechanism: the definition of the lemma as a hierarchical level sensitive to pseudomorphological decomposition. In AUSTRAL, the lemma level is organized hierarchically: the lemmas of “*play*” and “-*er*” are located at a subordinate level, such that they are activated directly from word-form, and they activate the superordinate lemma “*player.*” Importantly, morphological decomposition occurs not only with words carrying suffixes. So, words like “corner,” in which “-er” is a pseudosuffix, activate the “*corn,*” “-*er,*” and “*corner*” lemmas.

How could a dual-route mechanism be incorporated into AUSTRAL? We take into consideration that the reviewed evidence suggests that regular but not ambiguous nouns are decomposed during lexical access, and that gender cues are detected very early by comprehenders. Since the lemma is understood as an intermediate level that is sensitive to decomposition and captures correlations between form and function, it is possible to have a lemma for “-o” if it is correlated with a function. In particular, “-o” correlates with the masculine gender value in Spanish, since most masculine nouns end in “-o” (Harris, 1991). Therefore, we propose that a noun like “libro” (book) is first decomposed at the word-form level into LIBR- (as it is the stem included in many other words that share semantic features, such as “libreta” – “notebook” or “librería” – “bookstore”) and -O – which has been proposed to be a nominal classifier in Spanish (Fábregas, 2022).^1^

Form LIBR- would activate the lemma “*libr*,” and -O the lemma “-*o.*” The lemma “*libr*” would send activation to the superordinate lemma “*libro.*” Meanwhile,“-*o*” would send activation both to the lemma “*libro*” and to the masculine gender node. Note that the lemma “*libr*” by itself does not have gender (“libreta” and “librería” are feminine). Hence, the lemma “*libro*” would send activation to its semantic features plus the masculine node. This would implement a dual-route mechanism of gender retrieval: the lexical route, connecting the lemma “*libro*” to the masculine gender node, and the so-called form-based route, connecting the lemma “-*o*” to the masculine gender node. Since this route provides a pairing between a lemma representing sub-lexical information and the gender node, we call it the “sub-lexical route.” The direct connection between “-*o*” and the gender node would account for the fact that comprehenders detect gender regularities early during lexical access.

### Retrieval of gender with ambiguous and irregular nouns

Our review showed that ambiguous nouns do not seem to be morphologically decomposed and are less cognitively demanding than regular nouns. Hence, the gender node of ambiguous nouns should be activated through a lexical route. For example, an ambiguous Spanish noun like “raíz” (stem) would not have a decomposed lemma for “-z.” Fewer decomposition processes should translate into a smaller cognitive load.

In the case of irregular nouns, an inhibitory mechanism may be involved. For an irregular Spanish noun like “mano” (hand), which is feminine but ends in the prototypically masculine ending “-o,” decomposition would occur at the level of form between MAN- and -O (note that other words exist such as “manazas” (clumsy person), “manual” (manual), etc.). Yet, although “-o” correlates with the masculine gender value, it should not activate the masculine node. Spanish child learners in fact make gender assignment errors with irregular nouns (Figueira, 2001; Hernández-Pina, 1984; Pérez-Pereira, 1991). Since children have to correct themselves to master gender, and adult speakers successfully process the gender of these nouns, a mechanism of inhibition might be developed during gender acquisition.

Caffarra et al. (2015, 2017) obtained evidence of an early process of transparency in regular but not irregular nouns, with regular nouns showing greater amplitudes than irregular nouns in different electroencephalographic components. Thus, a mechanism of inhibition possibly operates early on during lexical access. Although the conflicting empirical evidence does not allow us to make conclusive statements about the specific nature of this inhibitory mechanism, we advance some hypotheses. Our starting point is the observation that children often make errors with irregular nouns, treating them as if they were regular. This suggests that children initially use a sub-lexical route to process irregular nouns, resulting in production errors. To successfully acquire gender, children need to learn how to stop the activation of the sub-lexical route. The outcome of the learning process is likely successful and results in the automatization of an inhibitory mechanism, which is why electroencephalographical evidence in Spanish and Italian adults does not suggest the involvement of a sub-lexical route in the processing of irregular nouns, with fMRI and behavioral studies pointing to the presence of an inhibitory mechanism.

An issue for future research is how to best describe the functioning and level of engagement of the inhibitory mechanism. First, since irregular nouns are often a small subset within a language, and children need exposure to identify them as irregular, word-form identification should be sufficient to trigger the inhibitory mechanism. Thus, one possibility is that the word-form representation of an irregular noun directly inhibits the sub-lexical route. Alternatively, since activation spreads from the lemma of the regular cue in parallel to the whole noun lemma and the gender node (as in “-*a*” to “*mapa*” [map, masculine Spanish noun even though it ends in “-a”] and to the feminine gender node), the whole noun lemma (“*mapa*”) could inhibit the sub-lexical route (although this would allow a bit of activation to accumulate at the “wrong” gender node, the feminine, previous to inhibition). Finally, perhaps the inhibitory mechanism works between lemmas “*map*-” and “-*a.*” Morphological decomposition would occur at the lemma level, but “*map*-” would inhibit “-*a*” very early, and hence lemma “*map*-” and form unit A would activate the whole lemma “*mapa.*”

### Activation and thresholds for selection: The role of articles

An interesting piece of evidence from gender-categorization tasks is that agreeing determiners seem necessary for gender retrieval with ambiguous nouns, irregular nouns, and regular nouns, at least in more opaque languages like French. The distinction between “activation” and “selection” in some models of language production (e.g., WEAVER++; Levelt et al., 1999) could help model this finding. The idea is that while gender nodes accumulate activation coming from the sub-lexical and lexical routes, they are only fully retrieved once an activation threshold has been reached. Thus, articles could be thought as a third source of gender activation along with the lexical and sub-lexical routes.

Articles are known to play a fundamental role in the acquisition of grammatical gender: children perceive articles and nouns as chunks of information, and statistical learning enables them to acquire grammatical gender and agreement (Arnon & Ramscar, 2012). Thus, articles and nouns might have a strong link in the adult mental lexicon. The literature on gender transparency supports this idea (see the section “Studies with explicit tasks”). Further, evidence by Taft and Meunier (Experiment 3, 1998) shows that, in grammaticality judgment tasks in French, facilitation is obtained for nouns that have an informative article, but not for those that have an uninformative one. This holds even when nouns are preceded by adjectives that start with a consonant and hence require informative definite articles.

To accommodate the findings above, we propose the existence of links between articles and nouns at the lemma level in a unidirectional way, from nouns to articles. Within the AUSTRAL model, the word-form LA in French (i.e., the feminine definite article) should be represented through the lemma "*la*" which represents definiteness, singular number, and feminine gender. On the other hand, the word-form L’ in French should be represented through the lemma “*l*’ ” which represents definiteness, singular number, and underspecified gender. A French noun like “classe” (class) would activate the definite article “la” whose lemma would be connected to the feminine gender node contributing to its activation. Meanwhile, a French noun like “école” (school) would activate the lemma for the definite article “*l*’ ” and facilitation during gender decision would not occur. Note that according to this rationale, other elements of agreement could be associated with nouns and display different degrees of activation depending on the frequency of their co-occurrence.

If the resting levels of activation of articles are high, they might be automatically recalled by participants to decide noun gender in experimental tasks. If articles that are informative of gender are not available, additional time would then be necessary to make gender decisions based on other agreement elements. If the degree of activation from the lexical and sub-lexical routes is enough for reaching the threshold of selection of a gender node, then an article would have a lesser role within a decision task, as likely happens in transparent languages with nouns ending in “-o” and “-a.”

### The role of the degree of gender transparency

The AUSTRAL model proposes that differently weighted connections between lemmas may exist, as well as different resting levels of activation depending on variables like frequency (Xu & Taft, 2015). The strength of the connections between lemmas varies continuously depending on the transparency of the relationship between the stem and its meaning (e.g., the strength of the connection is greater for “player” [“play-” + “-er”] than for words such as “archer” [“arch-” + “-er”]; Gonnerman et al., 2007; Xu & Taft, 2015). This means that the higher the semantic transparency between a stem and a suffix with the meaning, the stronger the connection between the stem lemma and the whole lemma for the noun, and hence the higher the degree of activation being sent from one to another.

The proposal above could be adopted to explain the role of gender transparency. Namely, the higher the degree of gender transparency of a language, the greater the strength of the links between the lemmas representing the gender regularities and their respective gender nodes. This would capture why a sub-lexical route has a prominent role during gender retrieval in transparent languages. However, some nuance is needed. Even in highly transparent languages, our review uncovered differences in gender retrieval between nouns displaying different types of regularities, as is the case in Spanish with nouns ending in “-o” and “-a” in comparison to nouns ending in (pseudo) suffixes such as “-ción” (Afonso et al., 2013). In short, results on gender transparency should take into account each type of regularity.

The results with regular nouns ending in “-o” and “-a” seem to be the most consistent and favorable to a sub-lexical route being used across different types of tasks. Indeed, “-o” and “-a” are present in approximately 60–70% of the Spanish and Italian nouns, and irregularity is rare, so the relationship between these cues and each gender value is particularly transparent. The higher the degree of transparency between a lemma and its functional gender node, the stronger their connections. Therefore, there might be a particularly strong connection between lemmas “-*o*” and “-*a*” and their corresponding gender nodes when compared to other regularities. Additionally, as “-o” and “-a” are very frequent, and since lemmas can have different resting levels of activation, “*-o*” and “*-a*” lemmas probably have higher resting levels of activation than the lemmas of other types of regularities that are not as frequent and common in the lexicon as these are. Both of these lemmas are hence maintained at a higher level of activation, and this activation spreads to the gender nodes themselves. Taken together, the strong links between lemmas and gender nodes and the higher resting levels of activation of the lemmas may underlie the abundant evidence of a sub-lexical route in Italian and Spanish.

### The role of the explicitness of a task

In our proposal a sub-lexical route is always used to retrieve the gender of regular nouns, but the links between lemmas’ regularities and gender nodes might vary in strength. In a categorization task, participants might strategically focus their attention on form cues, leading to more activation of the sub-lexical route. As a consequence, the mechanism of inhibition that was proposed for irregular nouns might fail (e.g., the word is not initially detected as irregular, inhibition does not occur for the sub-lexical route, or morphological decomposition is not inhibited), decreasing accuracy rates and increasing response times for irregular nouns (Padovani & Cacciari, 2003, Padovani et al., 2005). A similar reasoning could be applied to tasks involving agreement errors. When an agreement error is encountered, the form of nouns might engage the attention of participants who actively look for cues to detect grammatical errors and solve the task. Thus, the more explicit a task is, the more activated the sub-lexical route might be. Of course, the degree of transparency of each form of regularity should still affect the resting level of activation and strength of the connections between lemmas and gender nodes. This would explain why regularities like “-o/-a” show more consistent evidence of a sub-lexical route than other regularities, even under explicit conditions.

### The (lack of) interaction between word form regularity and noun frequency

Some experiments in French have found that higher frequency facilitates gender decisions, resulting in lower decision times for highly frequent nouns (Bulgarian: Andonova et al., 2004; French: Desrochers et al., 1989; Holmes & Segui, 2004, 2006; Muller-Gass et al., 2000; Taft & Meunier, 1998; but see Bates et al., 1995, for Italian). However, this frequency effect does not interact with the presence of regularities (Desrochers et al., 1989; Muller-Gass et al., 2000; Taft & Meunier, 1998; but see Desrochers et al., 1989, for an interaction on error rates). Based on our proposal, we would expect the degree of activation of a stem lemma – as well as that of the whole lemma – to influence the level of activation of the lexical route (Xu & Taft, 2015). For instance, in the case of a very frequent Spanish noun like "casa" (house) the subordinate and superordinate lemmas “*cas-*” and “*casa*,” respectively, would be highly activated. A highly activated superordinate lemma would send a high amount of activation through the lexical route. This would explain why highly frequent nouns allow faster gender decisions (Taft & Meunier, 1998). The lemma for “-*a*” is independent from the lemma “*cas-*,” so the amount of activation of “*cas-*” does not influence the amount of activation going from “-*a*” to the gender node through the sub-lexical route.

## Conclusions

We conducted a systematic review of studies on the role of noun-form regularities in gender processing, and we advanced a proposal of how to model such regularities within the AUSTRAL model of lexical access in comprehension. To experimentally validate this proposal, future research is necessary, especially with implicit tasks. Direct experimental comparisons between more and less transparent languages are critically needed to diagnose the role of form regularities cross-linguistically. Further, while several studies have used response-time paradigms like LDT and word naming, studies with a more finely grained temporal resolution like eye-tracking are still scarce. Finally, our review was limited to visual language comprehension because most studies have been conducted in this modality. Future studies using an aural presentation mode with words and sentences are necessary to examine whether our conclusions extend to other modalities.

## Data Availability

A list with details of each reviewed study is publicly available at https://osf.io/4kg6p.

## Footnotes

1 One possibility is that noun (pseudo)morphological decomposition occurs at the word-form level on the basis of the Basic Orthographic Syllabic Structure (BOSS; Taft, 1979, 2001). The BOSS divides a word by simultaneously maximizing the coda of the first subunit and considering relevant morphological aspects of the word. This would be particularly interesting for languages whose relationship with gender is not so clear-cut, such as French. In these languages, many monomorphemic nouns have sub-lexical units that are not systematically associated with a particular gender – i.e., the correlation is weaker than the Italian/Spanish “-o” and “-a” cues. By relying on the BOSS, in cases like French “-e” in a monomorphemic word like “classe” [class] and “-age” (stem + suffix “chômage” [unemployment]), the system could place a break after the BOSS (class + e; chôm + age), allowing (pseudo)decomposition with the gender cue: “-e” which correlates with the feminine, and “-age” which correlates with the masculine.”
